# Molecular detection and phylogenetic characterization of tick-borne pathogens in Anatolian water buffalo (*Bubalus bubalis*)

**DOI:** 10.1007/s11250-026-04890-5

**Published:** 2026-02-12

**Authors:** Nilgün Aydın, Neslihan Ölmez, Barış Sarı, Zati Vatensever, Gencay Taşkın Taşçı, Şemistan Kızıltepe, Mesut Erdi Işık, Mesut Yi̇ği̇t

**Affiliations:** 1 Department of Parasitology, Faculty of Veterinary Medicine, Kafkas University, Kars, 36100 Türkiye; 2 Tuzluca Vocational School, Iğdır University, Iğdır, 76100 Türkiye

**Keywords:** *Anaplasma*, *Babesia*, Incidental host, Molecular epidemiology, Spillover, *Theileria*

## Abstract

Tick-borne pathogens (TBPs) represent a major constraint to livestock health and productivity worldwide. Water buffaloes (*Bubalus bubalis*) are an important livestock species in Türkiye; however, molecular data on TBPs in this host remain limited. This study aimed to investigate the prevalence and molecular diversity of *Babesia*,* Theileria*,* Anaplasma*, and *Ehrlichia* species in water buffaloes from Iğdır Province, Eastern Türkiye. A total of 200 blood samples were analysed using genus-specific PCR assays targeting the 18S and 16S rRNA genes, followed by Reverse Line Blot (RLB) hybridisation and Sanger sequencing. Genus-level prevalence was 28.0% for *Theileria* spp., 6.0% for *Babesia* spp., and 2.5% for *Anaplasma* spp., while *Ehrlichia* spp. were not detected. Six species were identified: *Theileria annulata* (22.0%), *T. ovis* (5.0%), *T. buffeli* (1.0%), *Babesia canis* (5.0%), *B. ovis* (1.0%), and *Anaplasma phagocytophilum* (2.5%). This study provides the first molecular evidence of *B. canis* and *B. ovis* DNA in water buffaloes. However, the detection of parasite DNA alone does not confirm active infection and likely reflects incidental or spillover events associated with shared tick vectors in multi-host grazing systems. Phylogenetic analyses based on 18S and 16S rRNA genes revealed high sequence similarity with isolates from neighbouring regions, supporting regional circulation of closely related strains. These findings highlight the exposure of Anatolian water buffaloes to a broad spectrum of TBPs and underline the need for integrated studies combining vector surveillance, multilocus genotyping, and serological approaches to clarify host competence and transmission dynamics.

**Supplementary Information:**

The online version contains supplementary material available at https://doi.org/10.1007/s11250-026-04890-5.

## Introduction

Tick-borne diseases (TBDs) remain a major veterinary and economic concern in tropical and subtropical regions, where climatic and ecological conditions favor the proliferation of tick vectors and the pathogens they transmit (Jongejan and Uilenberg 2004). The geographical distribution and epidemiology of these diseases are largely determined by environmental factors affecting vector abundance, host availability, and pathogen–vector compatibility (Cardillo et al. 2025).

Among the most important tick-borne hemoparasites are protozoa of the genera *Babesia* and *Theileria* and rickettsial bacteria of the genera *Anaplasma* and *Ehrlichia* (Jongejan and Uilenberg 2004; Hussain et al. 2024; Khalifa et al. 2025). These pathogens infect domestic and wild ruminants and are associated with clinical manifestations such as fever, anemia, weight loss, reduced milk yield, and, in severe cases, mortality (OIE 2023; Şahin et al. 2023; Fatima et al. 2024). Subclinical and chronic infections in carrier animals play an important role in the maintenance of endemic transmission cycles, highlighting the need for continuous surveillance of tick-borne pathogens (Kocan et al. 2000; Şahin et al. 2022).

Water buffaloes (*Bubalus bubalis*) are valuable livestock species owing to their high productivity for milk and meat, adaptability to harsh environmental conditions, and relative tolerance to infectious diseases. In Türkiye, buffalo husbandry has gained renewed importance in recent years through government-supported breeding programs, resulting in a steady increase in the national buffalo population (Yılmaz et al. 2021; Şahin et al. 2023; Secato et al. 2025).

Iğdır Province, located in Northeastern Anatolia, is characterized by a unique microclimate that supports both agriculture and livestock production. The region’s geographical and ecological features favor the presence of diverse tick species (Değer et al. 2016; Taşçı et al. 2024; Afşar et al. 2025) and provide suitable conditions for buffalo farming (Yılmaz et al. 2021). Despite these favorable conditions, molecular and epidemiological data on tick-borne pathogens in water buffaloes from this region remain scarce.

Previous studies have demonstrated that buffaloes may harbor a variety of tick-borne pathogens, particularly *Theileria* spp. and *Babesia* spp., with considerable genetic diversity reported worldwide. Several *Anaplasma* species, including *A. marginale* and *A. phagocytophilum*, have also been detected in buffalo populations, although their epidemiological significance may vary among regions (El-Alfy et al. 2023; Cardillo et al. 2025). Nevertheless, comprehensive molecular investigations focusing on buffaloes in Türkiye are still limited.

Therefore, the present study was designed to detect and molecularly characterize tick-borne pathogens infecting water buffaloes (*Bubalus bubalis*) in Iğdır Province, Eastern Türkiye. The objectives were to determine the prevalence, molecular diversity, and phylogenetic relationships of *Babesia*, *Theileria*, *Anaplasma*, and *Ehrlichia* species using genus-specific PCR, Reverse Line Blot (RLB) hybridization, and Sanger sequencing analyses. This study provides the first molecular evidence of TBPs in water buffaloes from this region, contributing valuable data to the understanding of tick-borne pathogen ecology in Anatolian buffalo populations.

## Materials and methods

This study was conducted with the permission of Kafkas University Animal Experiments Local Ethics Committee (KAU-HADYEK/2022 − 155).

### Study area and sample collection

This study aimed to detect tick-borne hemoparasites (*Babesia*, *Theileria*, *Anaplasma*, and *Ehrlichia*) circulating in water buffaloes (*Bubalus bubalis*) in Iğdır Province, located on the eastern border of Türkiye, using molecular methods.

**Fig. 1:**
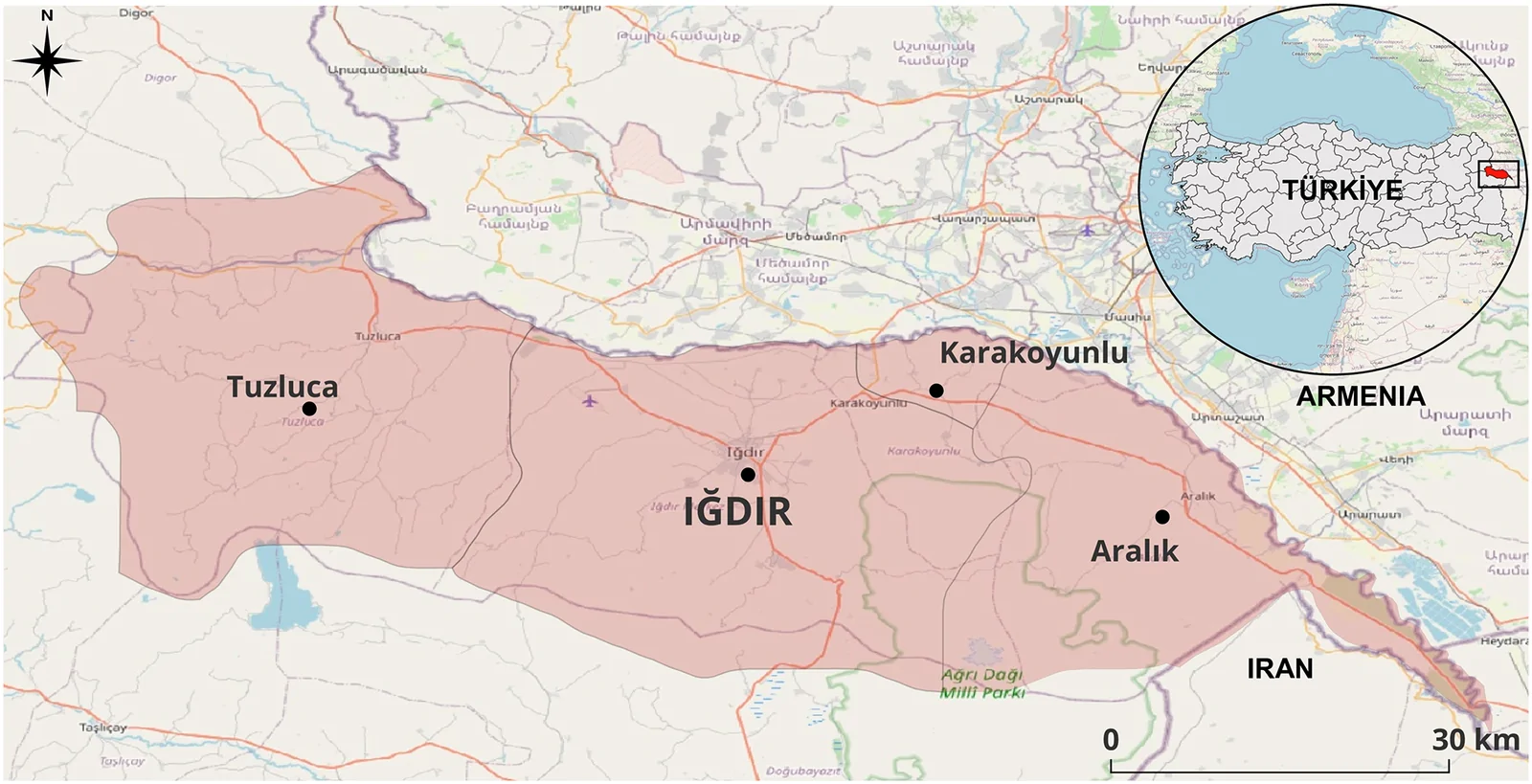
Map of Iğdır Province showing the sampling locations of water buffalo blood samples created using QGIS software (version 3.36, QGIS Development Team 2025)

Sampling was conducted between October 2022 and December 2024 at private and municipal slaughterhouses located in the Iğdır City Center (Fig. 1). Blood samples were collected during slaughter from 200 water buffaloes of different ages and sexes. Approximately 5 mL of blood was drawn aseptically from the jugular vein during exsanguination and transferred into EDTA-coated tubes. The samples were transported to the Parasitology Laboratory of Kafkas University under cold-chain conditions (4 °C) and stored at − 20 °C until DNA extraction. The general health status, age, and sex of each animal were recorded at the time of sampling.

Clinical health status was assessed at the time of slaughter based on general physical condition and the absence of overt clinical signs, as detailed clinical or longitudinal clinical follow-up was not feasible under slaughterhouse conditions.

### DNA extraction, molecular detection by PCR, and reverse line blotting (RLB)

Genomic DNA was extracted from whole blood using a commercial DNA extraction kit (EcoPURE Blood Genomic DNA Kit, Cat. No: E1075-50x, Türkiye) following the manufacturer’s instructions. Extracted DNA samples were stored at − 20 °C until molecular analyses were performed.

In the molecular part of the study, DNA extracted from blood samples was analyzed for the presence of *Babesia*, *Theileria*, *Anaplasma*, and *Ehrlichia* species using genus-specific primers (Table 1). PCR amplification was performed with primer pairs designed for the sensitive detection of these hemoparasites at the genus level. The RLBF2/RLBR2 (Georges et al. 2001) primer set targeting the 18S SSU rRNA V4 region was used for *Babesia/Theileria* detection, while the 16S8FE/BGA1B (Schouls et al. 1999) primer set targeting the 16S SSU rRNA V1 region was used for *Anaplasma/Ehrlichia* detection.

**Table 1:** Primers and species-specific oligonucleotide probes used in PCR and Reverse Line Blotting (RLB) assays

Primers	Sequences (5’-3’)	Reference
RLB-F2	GAC ACA GGG AGG TAG TGA CAA G	Georges et al. 2001
RLB-R2	biotin-CTA AGA ATT TCA CCT CTG ACAGT	
16S8FE	GGA ATT CAG AGT TGG ATC ATG GCT CAG	Schouls et al. 1999
B-GA1B	biotin-CGGGATCCCGAGTTTGCCGGGACTTCTTCT	
**Probes**	**Sequences (5’-3’)**	**Reference**
Catchall (*Theileria* spp.+*Babesia* spp.)	Amino-TAA TGG TTA ATA GGA(AG)C(AG)GTTG	Gubbels et al. 1999
*Theileria* genus specific	Amino-GTT GAA TTT CTG CT(A/G) CAT (C/T)GC	Nagore et al. 2004
*Theileria* spp.	Amino-TGA TGG GAA TTT AAA CC(CT)CTTCCA	Nagore et al. 2004
*Theileria annulata*	Amino-CCT CTG GGG TCT GTGCA	Georges et al. 2001
*Theileria buffeli/orientalis*	Amino-GGC TTA TTT CGG (AT)TTGATTTT	Gubbels et al. 1999
*Babesia* catchall 1	Amino-ATT AGA GTG TTT CAA GCA GAC	Adamu et al. 2014
*Babesia* catchall 2	Amino-ACT AGA GTG TTT CAA ACA GGC	Adamu et al. 2014
*Babesia* spp.	Amino-CCT(GT)GGTAATGGTTAATAGGAA	Schnittger et al. 2004
*Babesia bigemina*	Amino-CGT TTT TTC CCT TTT GTT GG	Gubbels et al. 1999
*Babesia bovis*	Amino-CAG GTT TCG CCT GTA TAA TTGAG	Georges et al. 2001
*Babesia major*	Amino-TCCGACTTTGGTTGGTGT	Georges et al. 2001
*Babesia divergens*	Amino-GTT AAT ATT GAC TAA TGT CGAG	Gubbels et al. 1999
Catchall (*Anaplasma* spp.+*Ehrlichia* spp.)	Amino-GGG GGA AAG ATT TAT CGC TA	Matjila et al. 2008
*Anaplasma centrale*	Amino-TCG AAC GGA CCA TAC GC	Matjila et al. 2008
*Anaplasma marginale*	Amino-GAC CGT ATA CGC AGC TTG	Matjila et al. 2008
*Anaplasma (E.) bovis*	Amino-GTA GCT TGC TAT GRG AAC A	Georges et al. 2001
*Anaplasma (E.) phagocytophilum* group	Amino-TTG CTA TAA AGA ATA ATT AGT GG	Bekker et al. 2002

Each PCR reaction was prepared in a final volume of 25 µL, containing 2.5 µL of DNA template, 1.25 µL of each primer (10 pmol/µL), 12.5 µL of 2× PCR Master Mix (EcoTaq), and 8.5 µL of nuclease-free water. Positive control DNA and distilled water (negative control) were included in each reaction set.

A Touchdown PCR protocol was employed to minimize non-specific amplification. For *Theileria* and *Babesia* species, initial denaturation was performed at 94 °C for 5 min, followed by denaturation at 94 °C for 15 s, annealing at 67 °C for 15 s, and extension at 72 °C for 30 s. The annealing temperature was decreased by 2 °C every two cycles until it reached 57 °C (67–65–63–61–59–57 °C). Subsequently, 40 cycles were performed at 94 °C for 1 min, 57 °C for 1 min, and 72 °C for 1 min, followed by a final extension at 72 °C for 5 min.

For *Anaplasma* and *Ehrlichia* species, the same conditions were applied, except that the annealing temperature started at 60 °C and decreased by 2 °C every two cycles until reaching 50 °C (60–58–56–54–52–50 °C). This was followed by 40 cycles at 94 °C for 1 min, 50 °C for 1 min, and 72 °C for 1 min, with a final extension at 72 °C for 5 min.

Five microliters of each PCR product were analyzed by electrophoresis on a 1.5% agarose gel stained with ethidium bromide and visualized under UV transillumination to confirm the expected amplicon sizes. The remaining 20 µL of each product was stored at + 4 °C for subsequent Reverse Line Blotting (RLB) analysis.

RLB is a two-step molecular assay consisting of PCR amplification followed by hybridization with species-specific oligonucleotide probes for species identification. The reverse primers were synthesized with a biotin label to enable detection. Species-specific oligonucleotide probes labeled with an N-terminal N-(trifluoroacetamidohexyl-cyanoethyl, N,N-diisopropylphosphoramidite [TFA])-C6 amino linker were synthesized according to previously published sequences. Hybridization was performed on Biodyne C membranes, using probe concentrations ranging from 200 to 900 pmol per 150 µL of hybridization mixture (Table 1). Membrane preparation, hybridization, and post-hybridization washing steps were conducted as previously described (Taşçı et al. 2024; Aydın et al. 2025). Chemiluminescent signal detection was carried out using the ChemiDoc™ MP Imaging System (Bio-Rad, USA), and the presence of positive signals was identified by the appearance of dark hybridization dots at probe-specific loci.

### Sequencing and phylogenetic analysis of positive samples

Because the amplicons generated by the RLBF2/RLBR2 and 16S8FE/BGA1B primer sets are relatively short and may contain mixed templates due to genus-level amplification, they are not optimal for reliable Sanger sequencing and phylogenetic inference. Accordingly, selected RLB-positive samples were re-amplified using genus- or species-specific PCR assays targeting longer fragments of the 18S–16S rRNA genes to generate high-quality amplicons suitable for sequencing and phylogenetic analysis (Allsopp et al. 1993; Chen et al. 1994; Casati et al. 2006; Kawahara et al. 2006; Oosthuizen et al. 2008). To obtain sequence data appropriate for phylogenetic interpretation, a stepwise PCR strategy was applied. Briefly, *Babesia ovis*,* B. canis*,* Theileria ovis*, and *T. annulata* were initially screened using the Nbab F/R primers (Oosthuizen et al. 2008) and subsequently confirmed with the BJ1/BN2 primers (Casati et al. 2006). *Theileria buffeli* was first detected using the Nbab F/R primers (Oosthuizen et al. 2008) and then amplified with the 989/990 primer pair (Allsopp et al. 1993). For *Anaplasma phagocytophilum*, initial amplification was performed with the genus-specific EC9/EC12A primers (Chen et al. 1994), followed by confirmation using the species-specific SSAp-F/SSAp-R primers (Kawahara et al. 2006).

Selected PCR- and RLB-positive samples were subjected to nucleotide sequencing of the 18S and 16S SSU rRNA genes for species confirmation and genetic characterization. Sanger sequencing of the purified amplicons was performed by Medsantek Inc. (Istanbul, Türkiye). The quality and accuracy of the chromatograms were assessed using BioEdit (version 7.2.5) software.

For each detected pathogen, a subset of RLB-positive samples was selected for sequencing. Among these, 3 of 44 samples positive for *T. annulata*, 1 of 5 for *(A) phagocytophilum*, 1 of 10 for *T. ovis*, 1 of 2 for *T. buffeli*, 3 of 10 for *(B) canis*, and 2 of 2 for *B. ovis* were successfully re-amplified and subjected to Sanger sequencing. Sample selection for sequencing was based on the availability of sufficient DNA quantity and quality, the presence of clear and high-quality PCR amplicons, and strong RLB hybridization signal intensity, and was not stratified according to age, sex, or other host-related variables.

The forward and reverse nucleotide sequences obtained from each primer were aligned with reference sequences available in GenBank using MEGA7 software (version 7.0.26), and consensus sequences were generated. Phylogenetic relationships among the obtained 18S and 16S rRNA sequences and related reference strains were inferred using the Maximum Likelihood (ML) method implemented in MEGA7 (Kumar et al. 2016). The most appropriate nucleotide substitution model was selected automatically based on the Bayesian Information Criterion (BIC), with either the General Time Reversible (GTR) model (Nei and Kumar 2000) or the Tamura–Nei model (Tamura and Nei 1993) applied depending on the dataset. Node support was assessed using 1,000 bootstrap replications, and the final phylogenetic trees were visualized and edited in MEGA7.

Consensus sequences obtained in this study were deposited in GenBank, and accession numbers were obtained. Additionally, the consensus sequences of positive samples were compared with homologous pathogen sequences deposited in GenBank using the BLAST (Basic Local Alignment Search Tool) algorithm to assess nucleotide similarity. The application of additional genetic markers for multi-locus sequencing was not feasible within the scope of the current project due to budgetary and technical constraints.

### Statistical analysis

Statistical analyses were performed using appropriate statistical software. Prevalence estimates and corresponding 95% confidence intervals (CIs) were calculated using the Wilson score interval method, which provides more accurate coverage than the Wald method, particularly for small sample sizes and low-prevalence data. Detailed statistical methods, including the calculation of prevalence estimates and 95% confidence intervals using the Wilson score method, are provided in the Supplementary Information.

Associations between pathogen detection status and categorical variables, including age group and sex, were evaluated using the χ² test. When expected cell counts did not meet the assumptions of the χ² test, Fisher’s exact test was applied. Animals were categorized into age groups based on their chronological age for age-related analyses. Age groups were defined as < 1 year (Group 1), 1–3 years (Group 2), and > 3 years (Group 3). A p value < 0.05 was considered statistically significant.

## Results

### Prevalence of tick-borne pathogens in water buffaloes

Out of the 200 water buffalo blood samples examined, 73 (36.5%) were positive for at least one tick-borne pathogen by Reverse Line Blot (RLB) hybridization. The overall prevalence of detected genera was *Theileria* spp. (28.0%), *Babesia* spp. (6.0%), and *Anaplasma* spp. (2.5%). No *Ehrlichia* species were detected in any of the analyzed samples (Table 2).

**Table 2:** Prevalence and distribution of tick-borne hemoparasites detected in water buffaloes from Iğdır Province, Türkiye, according to age and sex groups

Species	*N*	Age			Sex		Total
		1	2	+ 3	Male	Female	
		50	91	59	92	108	200
***A.phagocytophilum***	**Pos**	4	1	0	3	2	5
	**Prev (95% CI)**	8 (3.1-18.83)	1.09 (0.19–5.96)	0.0 (0.0-6.11)	3.26 (1.11–9.15)	1.85 (0.50–6.50)	2.5 (1.07–5.71)
	**P (χ² /Fisher)**	**0.014 (8.451)**			0.855 (0.033)		
***T.annulata***	**Pos**	5	25	14	21	23	44
	**Prev (95% CI)**	10.0 (4.34–21.6)	27.47 (19.35–37.41)	23.72 (14.69–35.97)	22.82 (15.44–32.38)	21.29 (14.63–29.93)	22.0 (16.81–28.23)
	**P (χ² /Fisher)**	0.052 (5.887)			0.929 (0.008)		
***T.ovis***	**Pos**	0	4	6	4	6	10
	**Prev (95% CI)**	0.0 (0.0-7.13)	4.39 (1.72–10.76)	10.16 (4.74–20.46)	4.34 (1.70-10.65)	5.55 (2.57–11.59)	5.0 (2.73–8.95)
	**P (χ² /Fisher)**	**0.049 (6.021)**			0.948 (0.004)		
***T.buffeli***	**Pos**	0	2	0	2	0	2
	**Prev (95% CI)**	0.0 (0.0-7.13)	2.19 (0.60–7.66)	0.0 (0.0-6.11)	2.17 (0.59–7.58)	0.0 (0.0-3.43)	1.0 (0.27–3.57)
	**P (χ² /Fisher)**	0.298 (2.420)			0.408 (0.684)		
***B.canis***	**Pos**	3	5	2	4	6	10
	**Prev (95% CI)**	6.0 (2.06–16.21)	5.49 (2.36–12.22)	3.38 (0.93–11.54)	4.34 (1.70-10.65)	5.55 (2.57–11.59)	5.0 (2.73–8.95)
	**P (χ² /Fisher)**	0.788 (0.474)			0.948 (0.004)		
***B.ovis***	**Pos**	0	1	1	1	1	2
	**Prev (95% CI)**	0.0(0.0-7.13)	1.09 (0.19–5.96)	1.69 (0.29–8.99)	1.08 (0.19–5.90)	0.92 (0.16–5.05)	1.0 (0.27–3.57)
	**P (χ² /Fisher)**	0.669 (0.802)			1.00 (0.00)		

**N** = number of samples, **Pos** = number of positive samples, **Prev (95% CI)** = prevalence with 95% confidence interval, **P (χ²)** = p-value obtained from the chi-square test. **Age groups**: Group 1 = < 1 year, Group 2 = 1–3 years, Group 3 = > 3 years. P values were calculated using χ² test; Fisher’s exact test was applied when expected cell counts were < 5

Among *Theileria* species, *T. annulata* was the most prevalent (22.0%; 44/200), followed by *T. ovis* (5.0%; 10/200) and *T. buffeli* (1.0%; 2/200). Within *Babesia* species, *B. canis* DNA was detected in 5.0% (10/200) and *B. ovis* DNA in 1.0% (2/200) of the samples. *Anaplasma phagocytophilum* was the only *Anaplasma* species identified (2.5%; 5/200).

To our knowledge, this represents the first molecular detection of *B. canis* and *B. ovis* DNA in water buffaloes.

All animals in which *B. canis* and *B. ovis* DNA was detected were clinically healthy at the time of sampling, with no overt clinical signs observed during routine ante-mortem inspection.

### Sequencing and phylogenetic findings

Selected PCR- and RLB-positive samples representing *A. phagocytophilum*, *T. annulata*, *T. ovis*, *T. buffeli*, *B. canis*, and *B. ovis* were subjected to Sanger sequencing for molecular confirmation and phylogenetic analysis. The obtained sequences were compared with homologous sequences available in GenBank using BLASTn analysis, revealing high nucleotide identity values (98–100%) with reference sequences.

Phylogenetic relationships were inferred using the Maximum Likelihood method based on partial 16–18S rRNA gene sequences. All sequences clustered within their respective species-specific clades together with reference isolates from different geographical regions. Sequences generated in this study are labelled as ‘this study’ and include the country of origin (Türkiye, Iğdır) and host species (water buffalo) within the sequence names.

### *Anaplasma phagocytophilum*

**Fig. 2:**
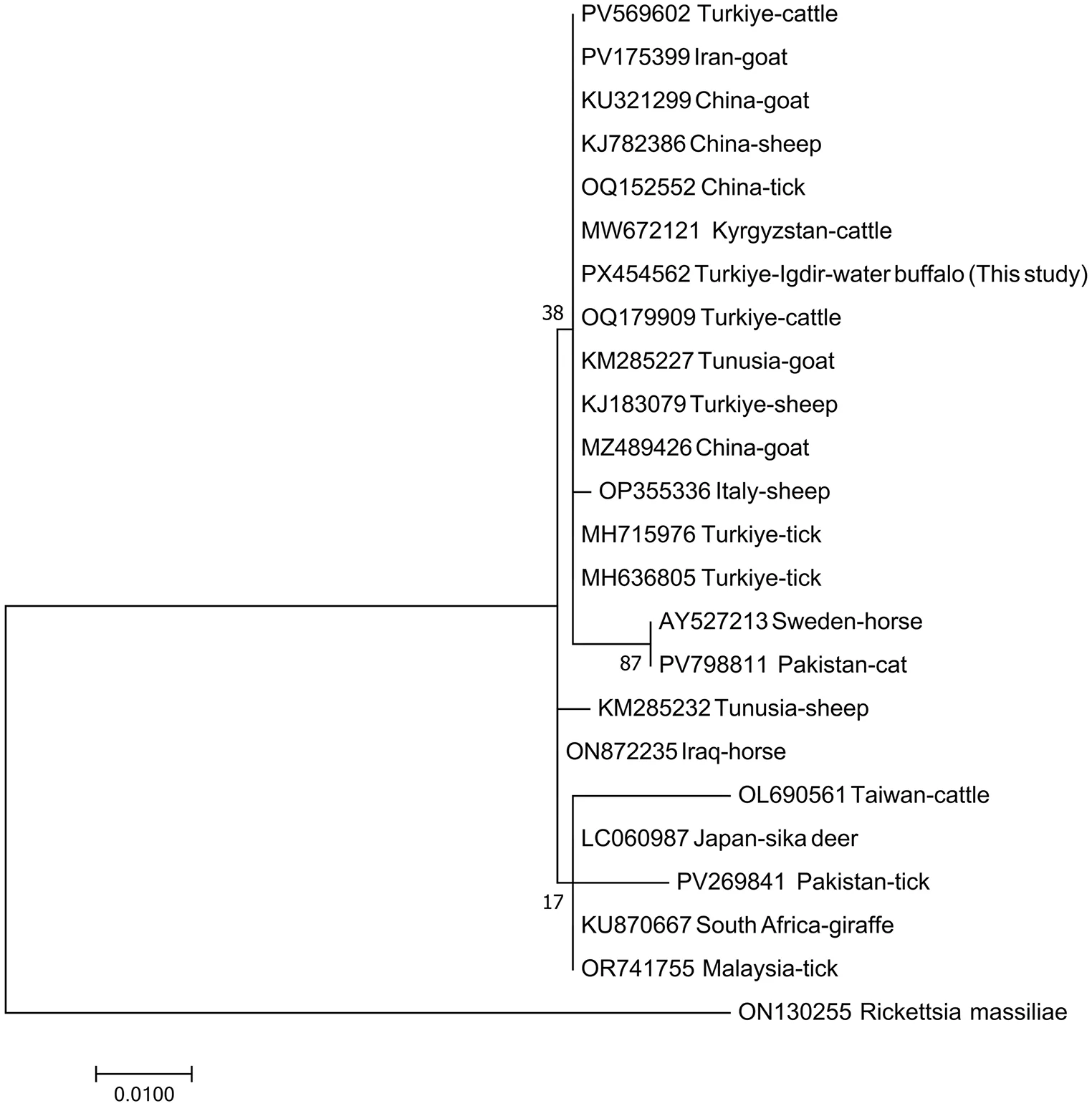
Molecular phylogenetic analysis of *Anaplasma phagocytophilum* based on partial 16S rRNA gene sequences inferred using the Maximum Likelihood method under the General Time Reversible model The tree with the highest log likelihood (− 2245.34) is shown and bootstrap values are indicated next to the branches The analysis included 24 nucleotide sequences and 1294 positions in the final dataset Evolutionary analyses were conducted in MEGA7 Sequences generated in this study are labelled as this study and include the country of origin (Türkiye, Iğdır) and host species (water buffalo)

The partial 16S rRNA gene sequence obtained from the *Anaplasma phagocytophilum* positive water buffalo was successfully amplified and sequenced. BLASTn analysis revealed a high nucleotide similarity (99.8–100%) with reference *A. phagocytophilum* sequences previously reported from ruminants and ticks in Türkiye, Iran, and China. Phylogenetic reconstruction using the Maximum Likelihood (ML) method based on the General Time Reversible (GTR) model (log likelihood = − 2245.34) demonstrated that the obtained sequence clustered within the *A. phagocytophilum* clade together with Eurasian reference isolates. The tree topology was supported by high bootstrap values, confirming the genetic conservation of the *A. phagocytophilum* 16S rRNA gene among ruminant-associated isolates (Fig. 2).

### *Theileria annulata*

**Fig. 3:**
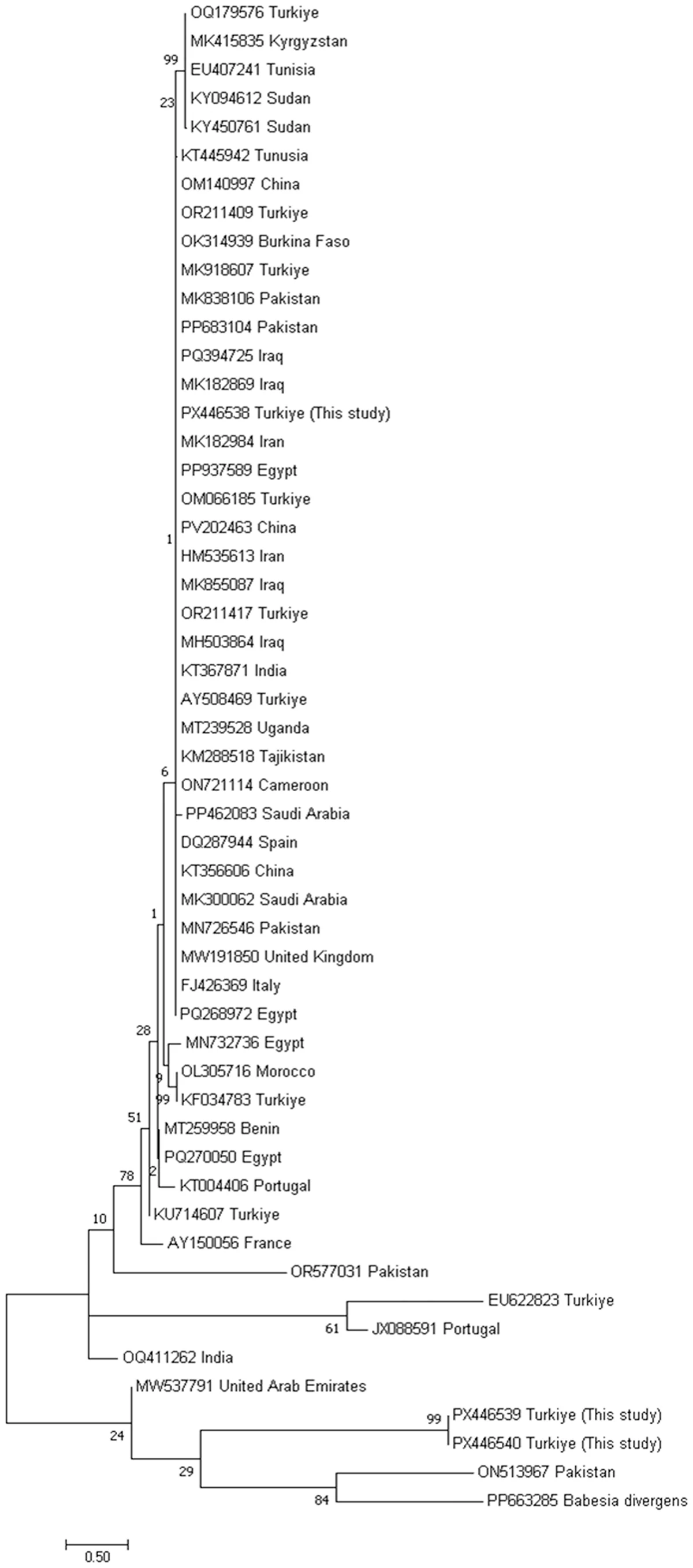
Molecular phylogenetic analysis of *Theileria annulata* based on partial 18S rRNA gene sequences inferred using the Maximum Likelihood method under the General Time Reversible model The tree with the highest log likelihood (− 6768.76) is shown and bootstrap values are displayed next to the branches The analysis included 53 nucleotide sequences and 457 positions Sequences generated in this study are labelled as this study and include the country of origin (Türkiye, Iğdır) and host species (water buffalo)

The *T. annulata* isolate clustered in a monophyletic group with reference sequences from cattle in Türkiye and neighboring countries. ML analysis based on the GTR model (log likelihood = − 6768.76) supported a conserved evolutionary lineage with strong bootstrap values (Fig. 3). This finding indicates close genetic relatedness between buffalo- and cattle-derived *T. annulata* isolates.

### *Theileria ovis*

**Fig. 4:**
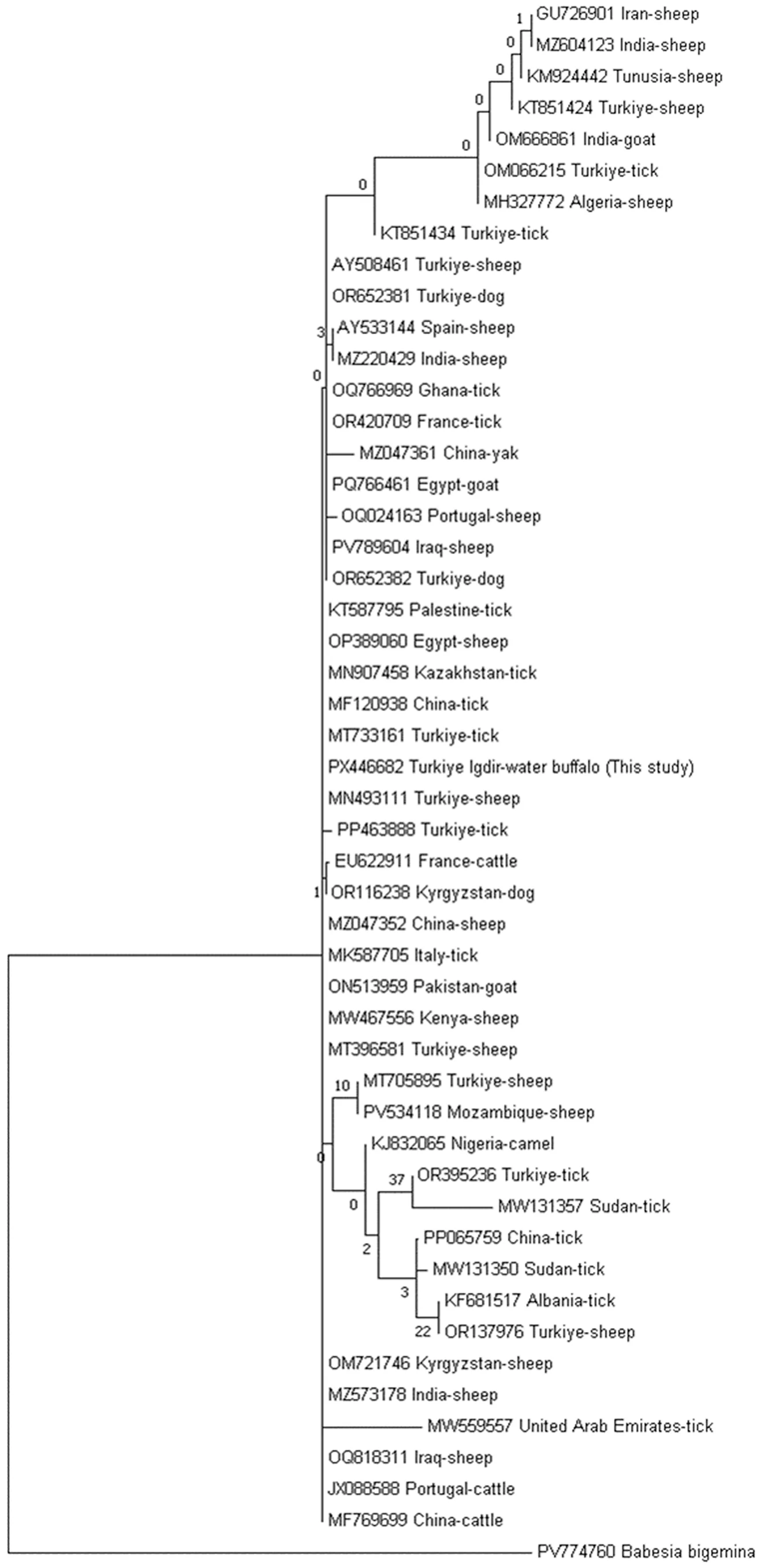
Molecular phylogenetic analysis of *Theileria ovis* based on partial 18S rRNA gene sequences inferred using the Maximum Likelihood method under the General Time Reversible model The tree with the highest log likelihood (− 3869.18) is shown and bootstrap values are indicated next to the branches The analysis included 50 nucleotide sequences and 1753 positions Sequences generated in this study are labelled as this study and include the country of origin (Türkiye, Iğdır) and host species (water buffalo)

The *T. ovis* sequence formed a well-supported clade with ovine isolates from Türkiye, Iran, and Egypt, exhibiting 99.8–100% nucleotide identity. ML analysis under the GTR model (log likelihood = − 3869.18) confirmed the genetic stability of *T. ovis* populations across ruminant hosts (Fig. 4).

### *Theileria buffeli*

**Fig. 5:**
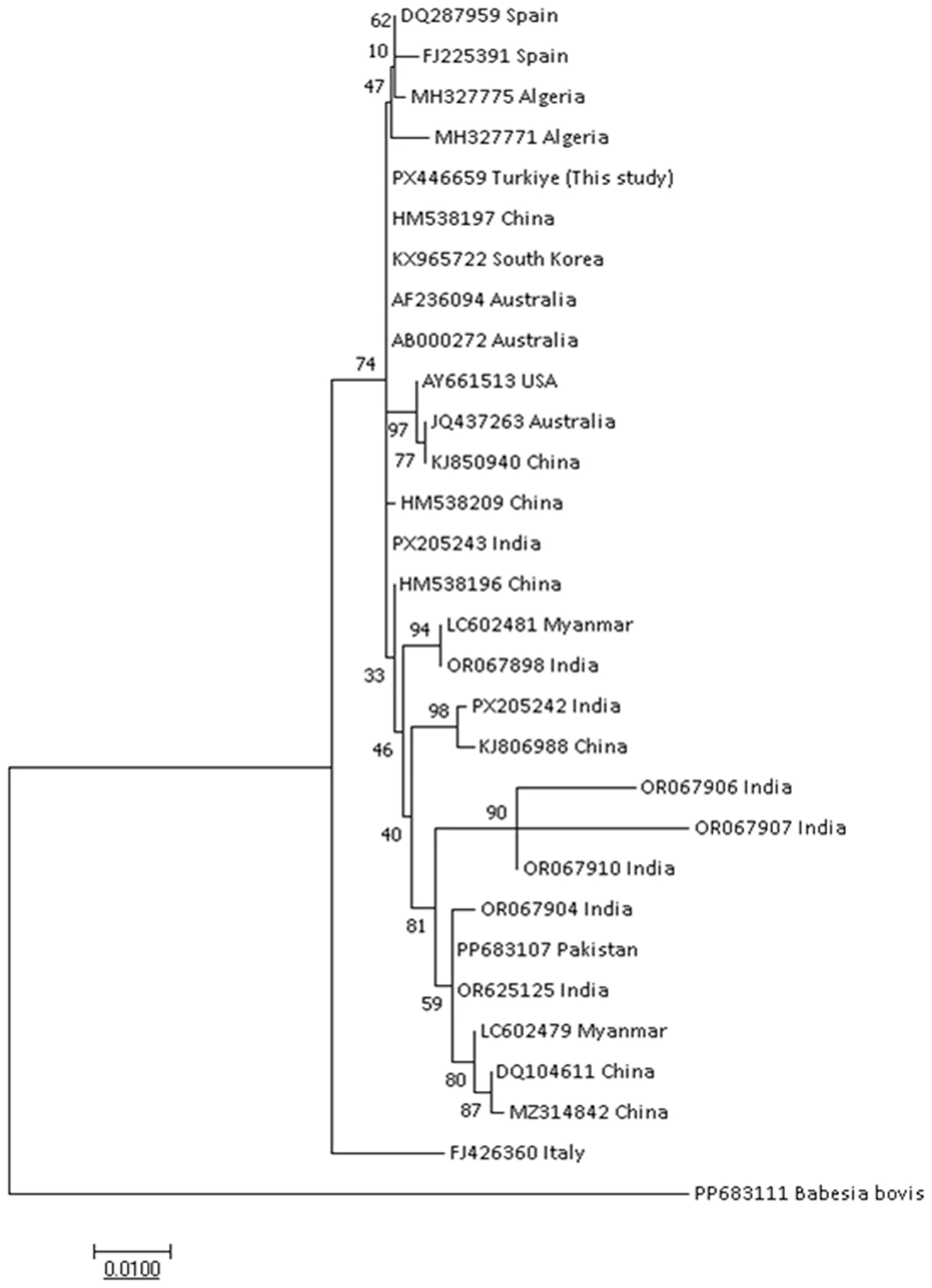
Molecular phylogenetic analysis of *Theileria buffeli* based on partial 18S rRNA gene sequences inferred using the Maximum Likelihood method under the Tamura–Nei model The tree with the highest log likelihood (− 2662.50) is shown and bootstrap values are indicated at the nodes The analysis included 30 nucleotide sequences and 1163 positions Sequences generated in this study are labelled as this study and include the country of origin (Türkiye, Iğdır) and host species (water buffalo)

Phylogenetic analysis of *T. buffeli* based on the 18S rRNA gene showed clustering with bovine isolates from China, India, and Türkiye, with 99.5–99.8% sequence identity. The ML tree constructed under the Tamura–Nei model (log likelihood = − 2662.50) placed the isolate within the *T. buffeli* clade with high bootstrap support (Fig. 5).

### *Babesia canis* and *Babesia ovis*

**Fig. 6:**
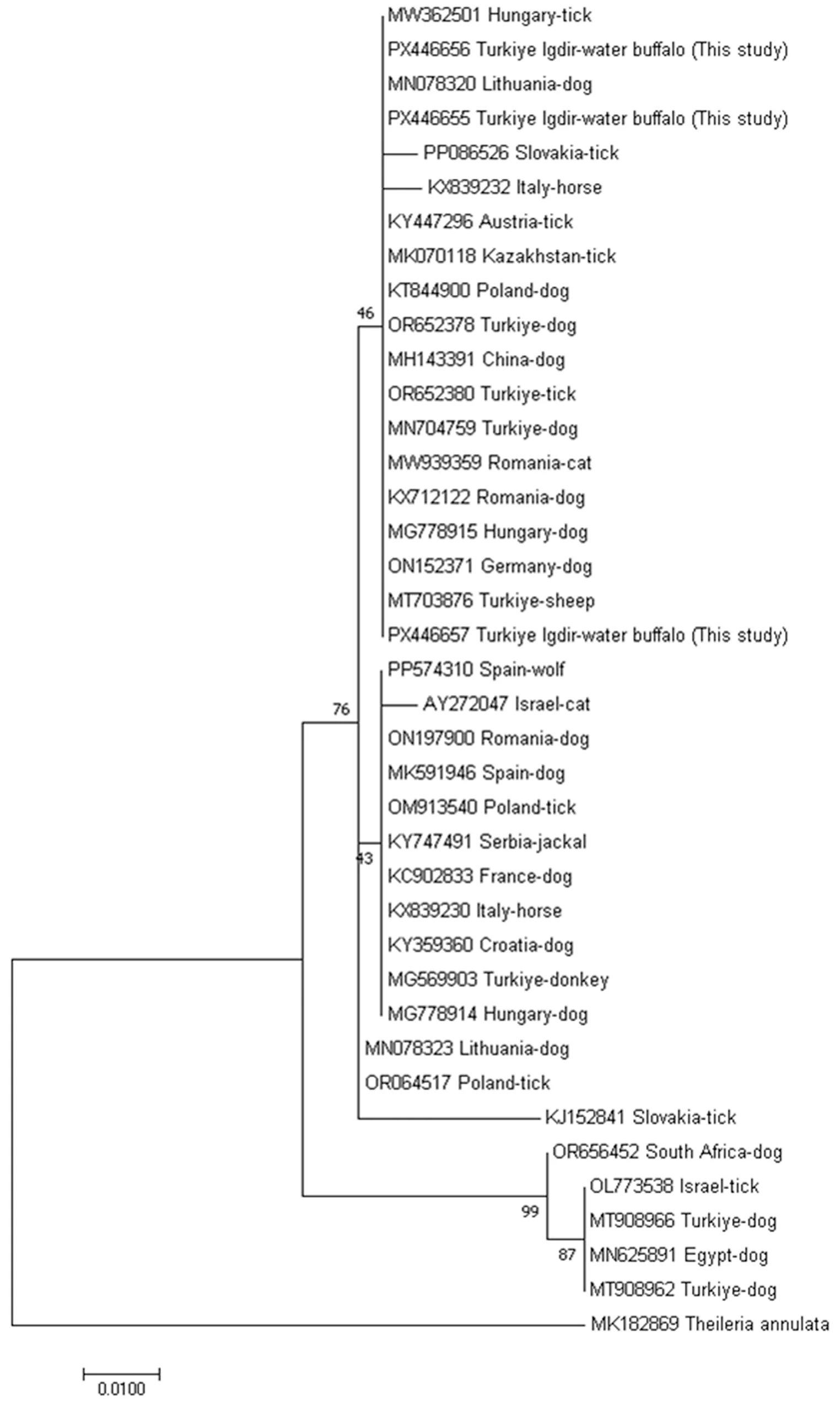
Molecular phylogenetic analysis of *Babesia canis* based on partial 18S rRNA gene sequences inferred using the Maximum Likelihood method under the Tamura–Nei model The tree with the highest log likelihood (− 1037.64) is shown and bootstrap values are displayed along the branches The analysis included 39 nucleotide sequences Sequences generated in this study are labelled as this study and include the country of origin (Türkiye, Iğdır) and host species (water buffalo)

**Fig. 7:**
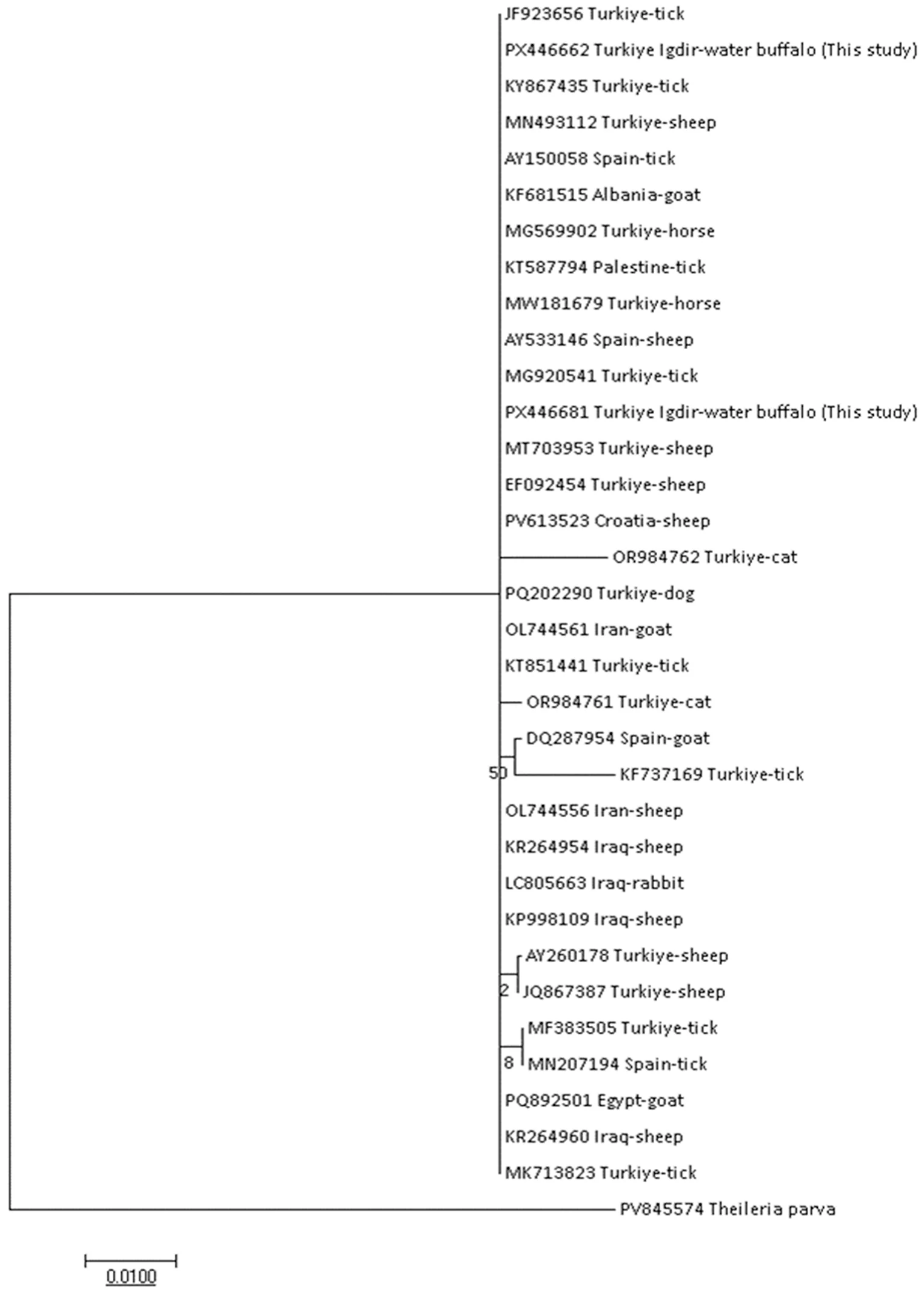
Molecular phylogenetic analysis of *Babesia ovis* based on partial 18S rRNA gene sequences inferred using the Maximum Likelihood method under the Tamura–Nei model The tree with the highest log likelihood (− 2011.68) is shown and bootstrap values are displayed next to the branches The analysis included 34 nucleotide sequences and 1271 positions Sequences generated in this study are labelled as this study and include the country of origin (Türkiye, Iğdır) and host species (water buffalo)

The *B. canis* isolate exhibited 99.7% similarity with *B. canis vogeli* sequences from dogs in Türkiye and Europe, while the *B. ovis* isolate showed 99.9% identity with ovine isolates from Türkiye and Iran. In the ML phylogenetic trees inferred under the Tamura–Nei model, both isolates clustered within their respective species clades with strong bootstrap support (Figs. 6 and 7). To our knowledge, this represents the first molecular evidence of *B. canis* and *B. ovis* DNA detection in water buffaloes.

All representative sequences were deposited to GenBank, and accession numbers were obtained (*A. phagocytophilum*: PX454562; *T. annulata*: PX446538–PX446540; *T. ovis*: PX446682; *T. buffeli*: PX446659; *B. canis*: PX446655–PX446657; *B. ovis*: PX446621, PX446681).

### Statistical results

The χ² test revealed significant associations between pathogen detection prevalence and age for *A. phagocytophilum* (*p* = 0.014) and *T. ovis* (*p* = 0.049). No statistically significant differences were observed with respect to sex for any of the detected pathogens (*p* > 0.05). Prevalence estimates and their corresponding 95% confidence intervals (CIs), calculated using the Wilson score interval method, are presented in Table 2. All animals included in the study were clinically healthy at the time of sampling; therefore, statistical analysis of health status in relation to pathogen detection could not be performed due to the absence of clinical variability within the study population.

## Discussion

The present study provides molecular and phylogenetic evidence for the detection of multiple tick-borne hemoparasites in Anatolian water buffaloes (*Bubalus bubalis*) from Eastern Türkiye. Using genus-specific PCR, Reverse Line Blot (RLB) hybridization, and Sanger sequencing, six species were identified: *Theileria annulata*,* T. ovis*,* T. buffeli*,* Babesia canis*,* B. ovis*, and *Anaplasma phagocytophilum*. The overall molecular detection rate (36.5%) indicates that buffaloes in the Iğdır region are frequently exposed to a broad spectrum of tick-borne agents, consistent with findings reported from neighboring provinces and bordering countries (Aktaş et al. 2005; Altay et al. 2014; Narimani et al. 2017; Aydın et al. 2022, 2025; Dakhil 2022; Kirman and Güven 2023). This observation likely reflects substantial tick exposure within mixed-host grazing systems; however, it should be interpreted with caution, as molecular detection alone does not permit definitive conclusions regarding active infection, sustained transmission cycles, or host reservoir competence.

According to a recent global meta-analysis, *Theileria* spp. are among the most frequently detected tick-borne hemoparasites in water buffaloes worldwide when assessed using the reverse line blot (RLB) assay (El-Alfy et al. 2023). Comparable detection patterns have been reported across Africa and Asia, although substantial heterogeneity exists among regions. Studies from endemic areas, including Pakistan, consistently identify *Theileria* annulata as the predominant species detected in buffalo populations (Khan et al. 2013). In the present study, *Theileria* spp. were commonly detected, with *T. annulata* representing the dominant species, while *T. ovis* and *T. buffeli* were identified less frequently. This pattern is consistent with the known epidemiology of *T. annulata* in endemic regions and likely reflects frequent exposure of buffaloes to infected tick populations. As noted above, molecular detection alone does not permit definitive conclusions regarding active infection or reservoir competence. Regional variation in reported detection rates is likely influenced by climatic conditions, livestock management practices, and the effectiveness of tick control measures, all of which shape tick abundance and host exposure risk.

Phylogenetic analyses based on the 18S rRNA gene indicate that the *Theileria buffeli* clade exhibits considerable genetic diversity and a broad geographical distribution and is often referred to as the *T. orientalis* complex, which remains taxonomically debated. Previous studies have documented the presence of *T. buffeli* DNA in buffalo populations from Asia and Africa (He et al. 2012; Chaisi et al. 2015), while *T. ovis* has been occasionally detected in buffaloes outside its classical small-ruminant host range (Abdullah et al. 2021). In the present study, the detection of *T. ovis* and *T. buffeli* DNA in buffaloes most likely reflects incidental exposure to infected ticks within multi-host grazing systems rather than established host–parasite relationships. Similar observations have been reported from northeastern Türkiye, where overlapping tick fauna were implicated in cross-species transmission events among sympatric ruminants (Taşçı et al. 2024). The clustering of buffalo-derived *T. ovis* and *T. buffeli* sequences with ovine and bovine isolates from Türkiye, Iran, and China is consistent with genetic conservation across hosts and suggests regional circulation of closely related *Theileria* lineages.

The detection of *Theileria ovis* DNA in buffaloes, a parasite typically associated with small ruminants, represents an unexpected finding. Partial 18S rRNA sequences amplified using the BJ1/BN2 primer set showed near-complete identity with reference *T. ovis* isolates deposited in GenBank and clustered firmly within the *T. ovis* clade, indicating close evolutionary relatedness to ovine strains. Closely related sequences have also been reported from ticks such as *Rhipicephalus turanicus* and *Hyalomma excavatum*, supporting the role of shared tick vectors in occasional cross-species transmission events. Nevertheless, the detection of *T. ovis* DNA in buffaloes is most plausibly explained by transient parasitemia or tick-borne spillover rather than a patent or epidemiologically significant infection.

It should be noted that species identification and phylogenetic inferences in the present study are based on single-locus (18S rRNA) data, which may limit resolution among closely related *Theileria* taxa. The inclusion of additional genetic markers (e.g. cytb, hsp70, cox1, and MPSP) was not feasible within the scope of the current project due to budgetary and technical constraints; therefore, future studies with dedicated funding will be required to further improve phylogenetic resolution and clarify host range and transmission dynamics (D’Oliveira et al. 1995; Sivakumar et al. 2014; Afshari et al. 2021).

The detection of atypical tick-borne pathogens in buffaloes can most plausibly be explained by spillover events occurring in multi-host grazing systems where buffaloes, cattle, small ruminants, and dogs share overlapping tick fauna. In the study area, tick species such as *Rhipicephalus bursa*,* R. turanicus*,* Hyalomma excavatum*, and *R. sanguineus* are commonly reported on domestic ruminants and have also been documented to parasitize water buffaloes opportunistically (Değer et al. 2016; Taşçı et al. 2024; Afşar et al. 2025). Among these, *R. turanicus* and *H. excavatum* are recognized as generalist ticks with broad host ranges and have been implicated in the transmission of *Theileria*,* Babesia*, and other tick-borne pathogens across different host species (Jongejan and Uilenberg 2004; Cardillo et al. 2025). These ecological conditions provide a plausible route for incidental exposure of buffaloes to pathogens typically associated with cattle, small ruminants, or dogs rather than evidence of established host–pathogen adaptation. However, determining whether buffaloes can support onward transmission or act solely as incidental or dead-end hosts requires further investigation. Future studies integrating targeted tick infestation surveys on buffaloes, molecular xeno-monitoring of attached ticks, longitudinal serological assessments, experimental transmission trials, and multi-locus genetic typing will be essential to clarify the epidemiological role of buffaloes within multi-host tick–pathogen systems.

Among *Babesia* species, *B. bovis* and *B. bigemina* are the most frequently reported in Asian water buffaloes and are primarily transmitted by *Rhipicephalus (Boophilus)* ticks. Less commonly detected species, including *B. orientalis*,* B. occultans*, and B. *naoakii* (formerly *Babesia* sp. *Mymensingh*), have also been reported in buffalo populations (El-Alfy et al. 2023). In the present study, *Babesia* DNA was detected at a relatively low frequency, with the identification of *B. canis* and *B. ovis* representing the first molecular evidence of these species in water buffaloes. Sequence analysis of partial 18S rRNA gene fragments showed near-complete identity with reference *B. canis* and *B. ovis* sequences deposited in GenBank, and phylogenetic reconstruction placed both isolates within their respective species-specific clades together with canine and ovine reference strains from neighboring regions.

Given the well-established host specificity of *Babesia canis* and *B. ovis*, their detection in buffalo blood is most plausibly explained by incidental exposure or tick-borne spillover rather than patent infection or sustained host adaptation. Such spillover events are likely facilitated by overlapping tick fauna in multi-host grazing systems, particularly generalist *Rhipicephalus* species such as *R. bursa* and *R. sanguineus*, which are known to parasitize multiple mammalian hosts. Nevertheless, molecular detection alone does not allow conclusions regarding active infection or transmission competence. It should therefore be emphasized that species assignment and phylogenetic interpretation in this study are based on single-locus (18S rRNA) data. Future studies incorporating additional genetic markers, including ITS1/ITS2, cytb, cox1, cox3, and hsp70, would be valuable to improve taxonomic resolution and to further clarify host range and transmission dynamics of *Babesia* species in multi-host ecosystems (Matjila et al. 2004; Yamasaki et al. 2007; Schnittger et al. 2012; Yabsley and Shock 2013; Uluçeşme et al. 2024).

The detection of *Anaplasma phagocytophilum* DNA in water buffaloes provides additional insight into the occurrence of this pathogen in an atypical host species. To date, clinical infection has been reported only sporadically in buffaloes, including a single confirmed case from Switzerland (Langenwalder et al. 2019). Comparative studies indicate marked regional variation in detection patterns, with higher occurrence reported in Italian buffalo populations and absence of detection in some Central European countries (El-Alfy et al. 2023; Cardillo et al. 2025). In Türkiye, *A. phagocytophilum*-like variants have previously been identified in domestic ruminants (Şahin et al. 2023).

In the present study, *A. phagocytophilum* was detected at a low frequency, suggesting that water buffaloes in the study area are likely exposed only sporadically to infected ticks. The lower detection rate compared with sympatric cattle populations from the same region, where higher prevalence has been reported (Aydın et al. 2025), further supports the interpretation that buffaloes may play a limited epidemiological role in the maintenance of *A. phagocytophilum*. Phylogenetic analysis based on partial 16S rRNA sequences revealed close clustering with Eurasian reference isolates from Türkiye, Iran, and China, indicating strong genetic homogeneity across strains. Age-related associations observed for *A. phagocytophilum* and *T. ovis* are consistent with cumulative tick exposure and host immunity patterns previously described in bovine anaplasmosis and theileriosis, whereas the absence of sex-related differences aligns with earlier reports in domestic ruminants (Bharti et al. 2022; Şahin et al. 2023).

The observed association between host age and the detection of *Anaplasma phagocytophilum* and *Theileria ovis* in the present study, particularly among animals older than three years, is more likely to reflect age-related differences in exposure dynamics rather than differences in clinical susceptibility. Older buffaloes generally experience longer grazing periods and cumulative contact with tick-infested environments compared with younger age groups (< 1 year and 1–3 years), which may increase the likelihood of pathogen DNA detection over time. Similar age-related patterns have been widely reported for tick-borne pathogens in ruminants, where variations among age groups are commonly attributed to prolonged exposure to infected ticks, grazing practices, and age-dependent modulation of host immune responses (Jongejan and Uilenberg 2004; Stuen et al. 2013). These findings support the interpretation that the observed age-related differences primarily reflect cumulative exposure rather than age-specific disease susceptibility.

The absence of overt clinical signs in all sampled buffaloes provides additional context for interpreting the molecular findings of this study. The lack of observable clinical correlations, particularly in animals positive for atypical pathogens such as *Babesia canis*,* B. ovis*,* Theileria ovis*, and *Anaplasma phagocytophilum*, supports the interpretation that these detections most likely represent incidental carriage or transient exposure rather than clinically relevant or productive infections. In the absence of clinical disease, serological evidence, or longitudinal follow-up, the detection of pathogen DNA alone should be interpreted cautiously, reinforcing the view that water buffaloes in the study area are more likely incidental or dead-end hosts for certain tick-borne pathogens rather than primary contributors to disease transmission cycles.

No *Ehrlichia* species were detected in the present study, which is consistent with previous findings reported in cattle from the same region (Aydın et al. 2025). The absence of *Ehrlichia* DNA in buffaloes may be related to regional differences in tick fauna, particularly the apparent scarcity of competent vectors such as *Amblyomma* spp., which are known to play a key role in the biological transmission of several *Ehrlichia* species. Although *Amblyomma* ticks were not observed during the present sampling period, the lack of detection should be interpreted cautiously, as vector presence can vary spatially and seasonally. Nevertheless, these observations suggest that regional vector composition and environmental conditions may influence the local transmission dynamics of *Ehrlichia* spp. and highlight the importance of broader and systematic tick surveillance to better assess the epidemiological risk in the area.

In conclusion, unlike previous reports that primarily identified well-known *Babesia*,* Theileria*, and *Anaplasma* species in water buffaloes (Prado et al. 2022; El-Alfy et al. 2023), the present study documents the molecular detection of atypical tick-borne hemoparasite DNA, including *B. canis*,* B. ovis*, and *T. ovis*, in buffaloes from Eastern Türkiye. Although global research on tick-borne pathogens in buffalo populations has expanded in recent years (Khan et al. 2025; Sansamur et al. 2025; Mahmoud et al. 2024; Cardillo et al. 2025), molecular data from Türkiye remain limited (Şahin et al. 2022, 2023). The findings of this study therefore contribute novel molecular evidence to the epidemiological characterization of tick-borne hemoparasites in Anatolian water buffaloes. Ecological factors such as shared pastures, overlapping tick fauna (*Rhipicephalus bursa*,* R. turanicus*,* Hyalomma excavatum*), and multi-host grazing systems are likely to facilitate transient DNA spillover among sympatric livestock species rather than indicating established host–parasite adaptation. Accordingly, further investigations integrating multilocus genetic typing, vector xeno-monitoring, experimental transmission studies, and longitudinal serological surveillance are required to distinguish incidental detections from biologically and epidemiologically relevant infections. Overall, this study enhances current knowledge of tick–pathogen–host interactions in water buffaloes from Eastern Türkiye. These findings highlight the complexity of tick-borne pathogen ecology in multi-species livestock systems.

## Conclusion

This study provides novel molecular and phylogenetic evidence for the detection of multiple tick-borne hemoparasites in Anatolian water buffaloes (*Bubalus bubalis*) from Eastern Türkiye. Using genus-specific PCR, Reverse Line Blot (RLB) hybridization, and Sanger sequencing, six species were identified: *Theileria annulata*,* T. ovis*,* T. buffeli*,* Babesia canis*,* B. ovis*, and *Anaplasma phagocytophilum*. The molecular detection of atypical *Babesia* species (*B. canis* and *B. ovis*) represents, to our knowledge, the first molecular evidence of their DNA in buffaloes and is consistent with sporadic spillover events facilitated by overlapping tick populations (e.g. *Rhipicephalus bursa*,* R. sanguineus*) and shared grazing environments. Overall, the findings support the interpretation that water buffaloes are more likely incidental or dead-end hosts rather than primary reservoirs for certain tick-borne pathogens within multi-host ecosystems.

The high genetic similarity between buffalo-derived isolates and reference sequences from neighboring regions suggests possible regional gene flow and vector sharing among sympatric ruminants. Future investigations integrating molecular xeno-monitoring of ticks, multilocus genetic typing, and longitudinal serological surveillance will be essential to better characterize transmission dynamics and to clarify the epidemiological relevance of tick-borne hemoparasites in Eastern Türkiye. Such integrated approaches may contribute to improved regional control strategies and a more refined understanding of pathogen circulation in mixed-host livestock systems.

## Supplementary Information

Below is the link to the electronic supplementary material.


Supplementary Material 1


## Data Availability

Data is available upon reasonable research purpose request to the corresponding author.
