# Supplementary material for: Molecular detection and phylogenetic characterization of tick-borne pathogens in Anatolian water buffalo (Bubalus bubalis)
Source: Trop Anim Health Prod. 2026 Feb 12;58(2):106. doi: 10.1007/s11250-026-04890-5 (PMC12901143; doi:10.1007/s11250-026-04890-5)
Supplement: Supplementary file 1 — Supplementary Material 1 [file 11250_2026_4890_MOESM1_ESM.docx]

**Supplementary Material – Statistical Methods**

**Statistical Analysis**

The prevalence rates of infections in individual samples and their 95% confidence intervals (CIs) were calculated using statistical methods based on the binomial distribution. The Wilson score interval method was preferred for confidence interval estimation, as the classical Wald method often yields unreliable results with small sample sizes or extreme prevalence values. Although the Wilson method is derived using a normal approximation framework, it incorporates a correction term, it introduces a correction term to the point estimate, providing confidence intervals with coverage probabilities closer to the nominal level, especially for small samples (Brown et al. 2001).

All calculations were performed using the R statistical software (R Core Team, version 4.3.2), and the “binom” package (Dorai-Raj 2014) was used for computing confidence intervals. For each pathogen, the number of positive samples (x) and the total number of samples (n) were considered, and the prevalence rate was calculated as p̂ = x/n. The 95% confidence interval according to the Wilson method was calculated using the following formula:

$$\frac{\hat{p}+\frac{z^{2}}{2n}\pm z\sqrt{\frac{\hat{p}(1-\hat{p})}{n}+\frac{z^{2}}{4n^{2}}}}{1+\frac{z^{2}}{n}}$$

where p̂ represents the observed prevalence, n the total sample size, and z the critical value from the standard normal distribution (e.g., 1.96 for a 95% confidence interval).

**Reference**

Brown LD, Cai TT, DasGupta A (2001) Interval estimation for a binomial proportion. Stat Sci 16(2):101–133

Dorai-Raj S (2014) binom: Binomial confidence intervals for several parameterizations. R package version 1.1-1. Available at: https://cran.r-project.org/package=binom
